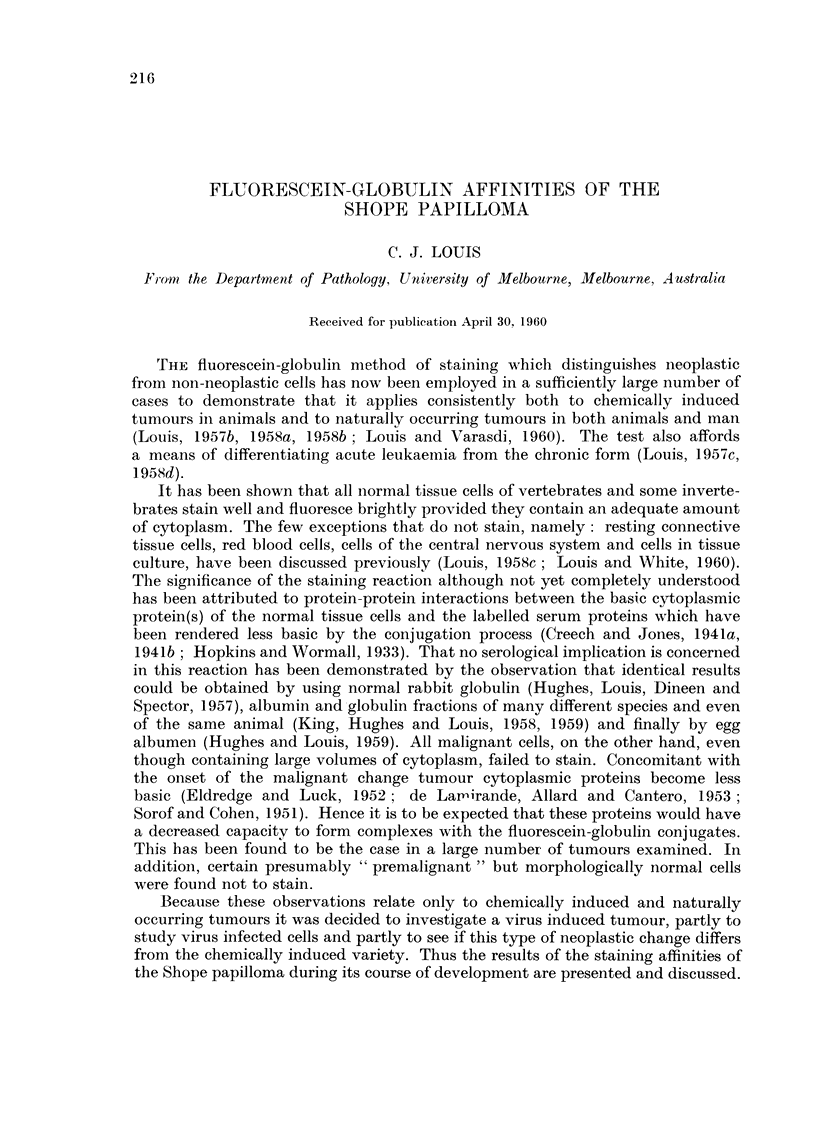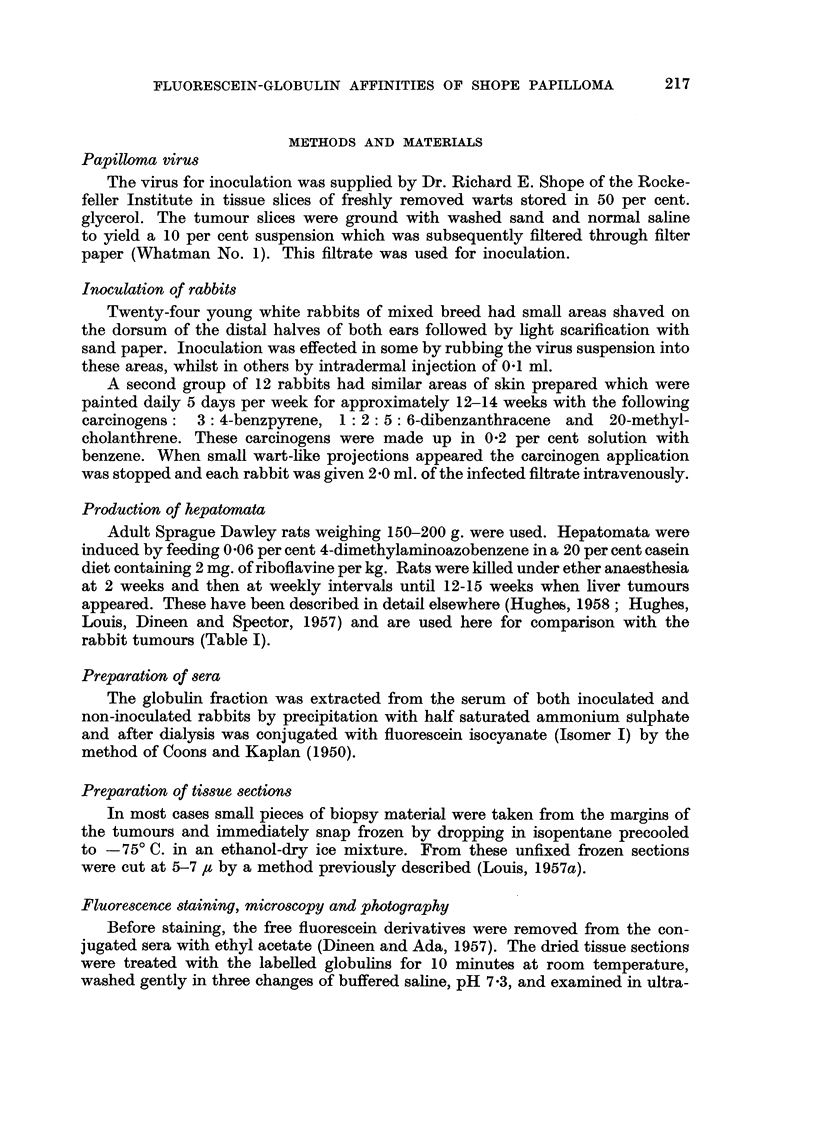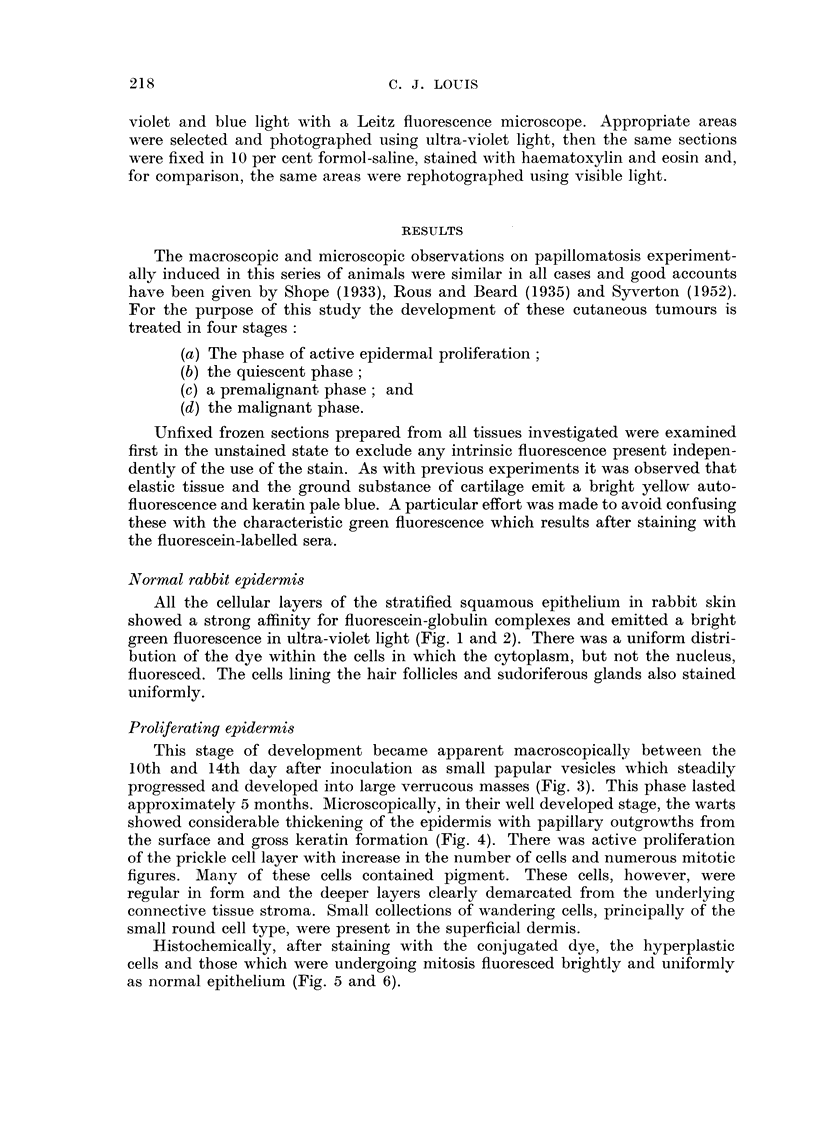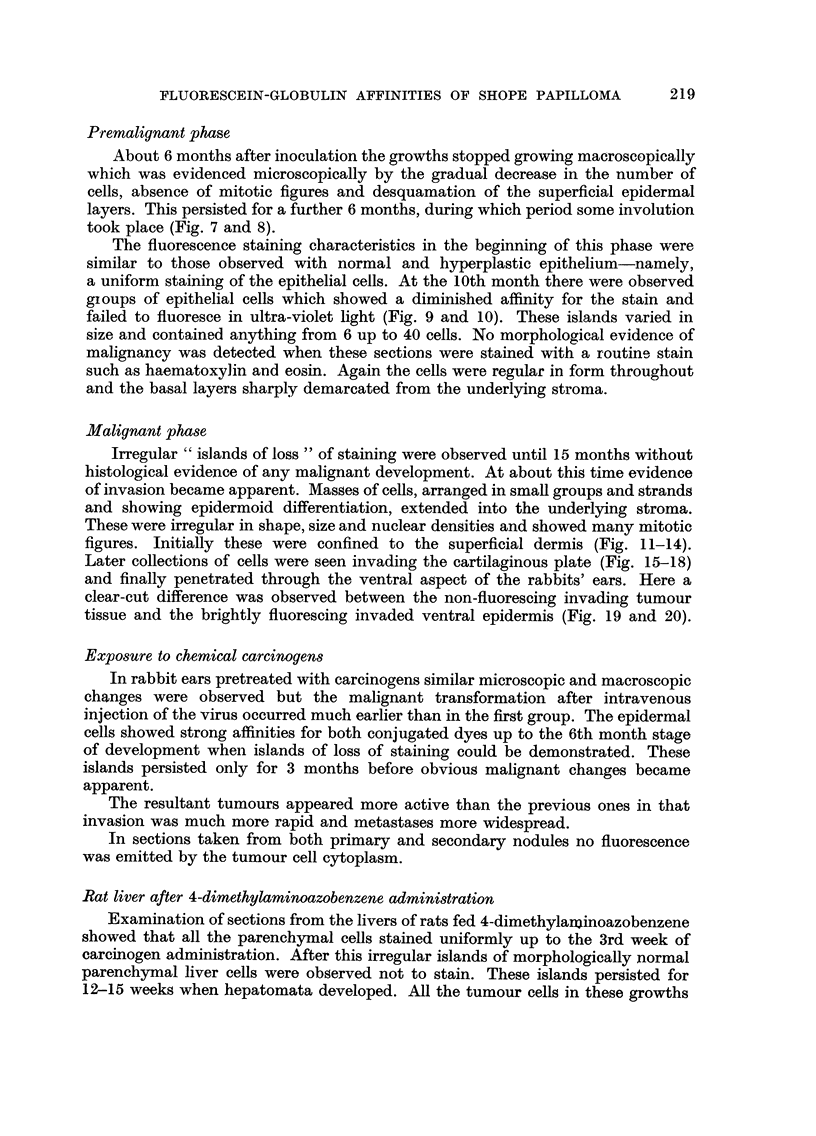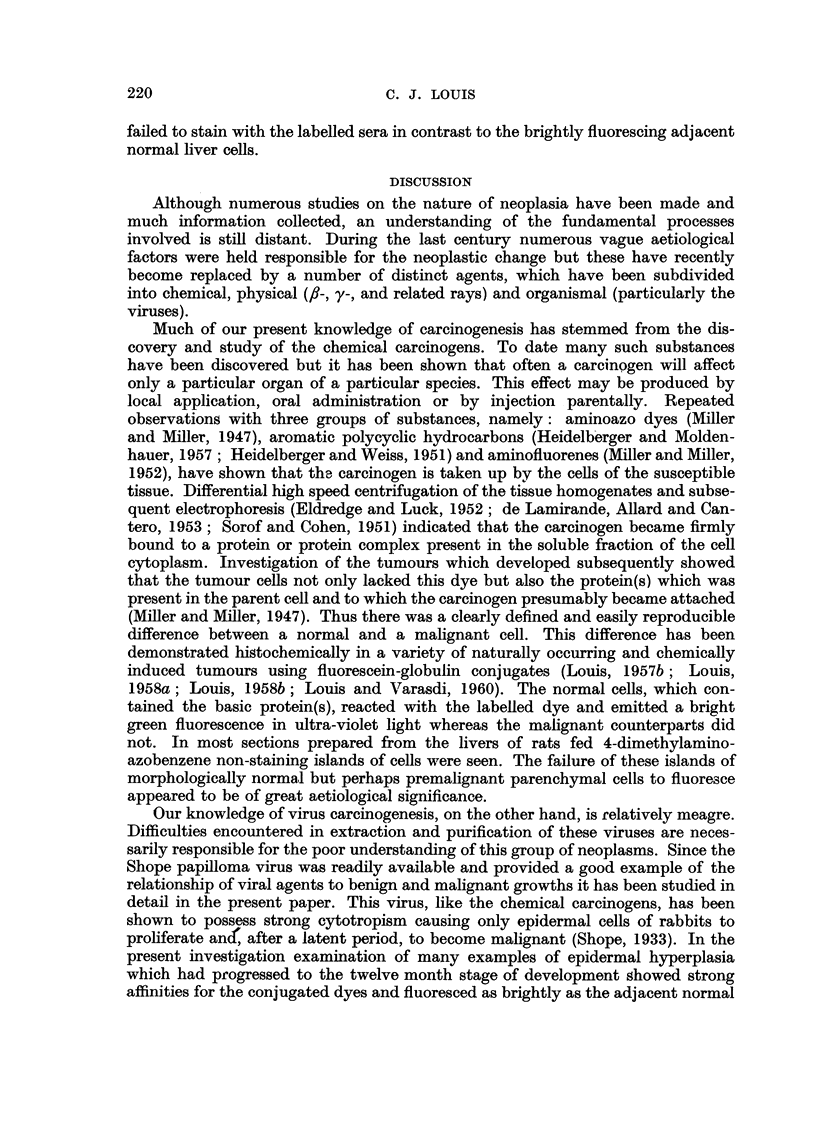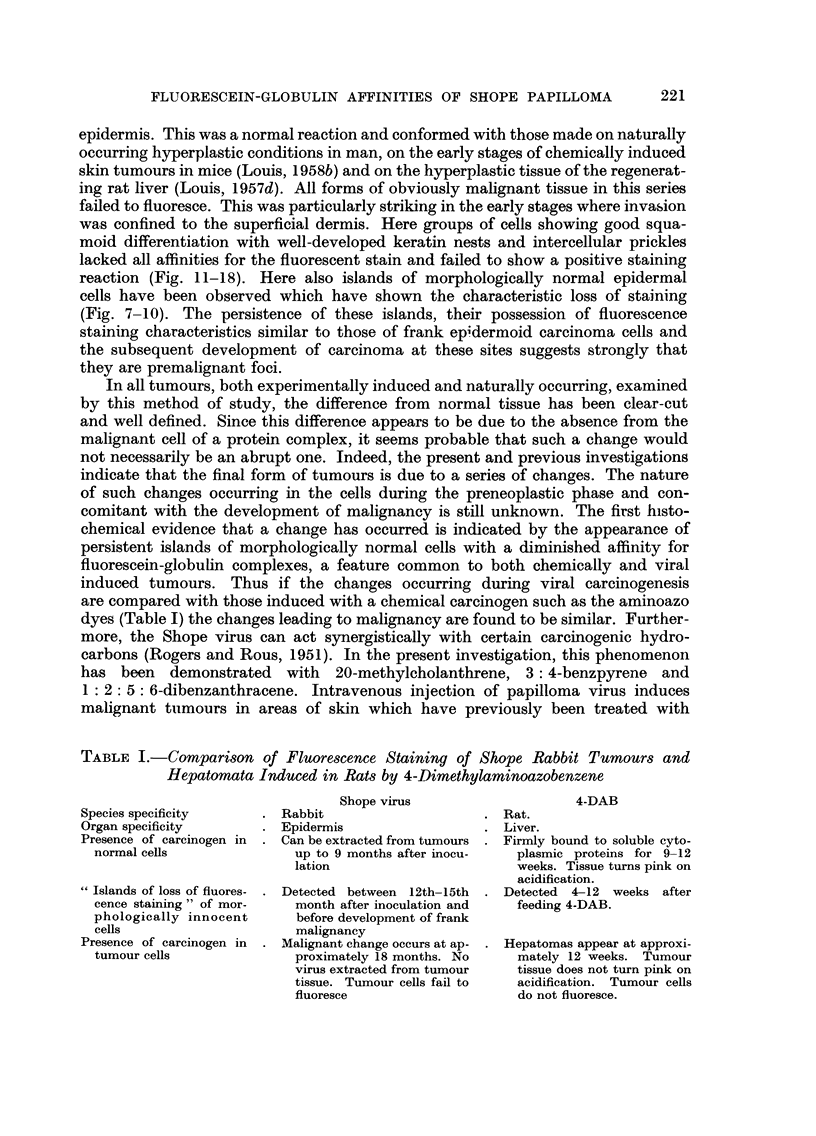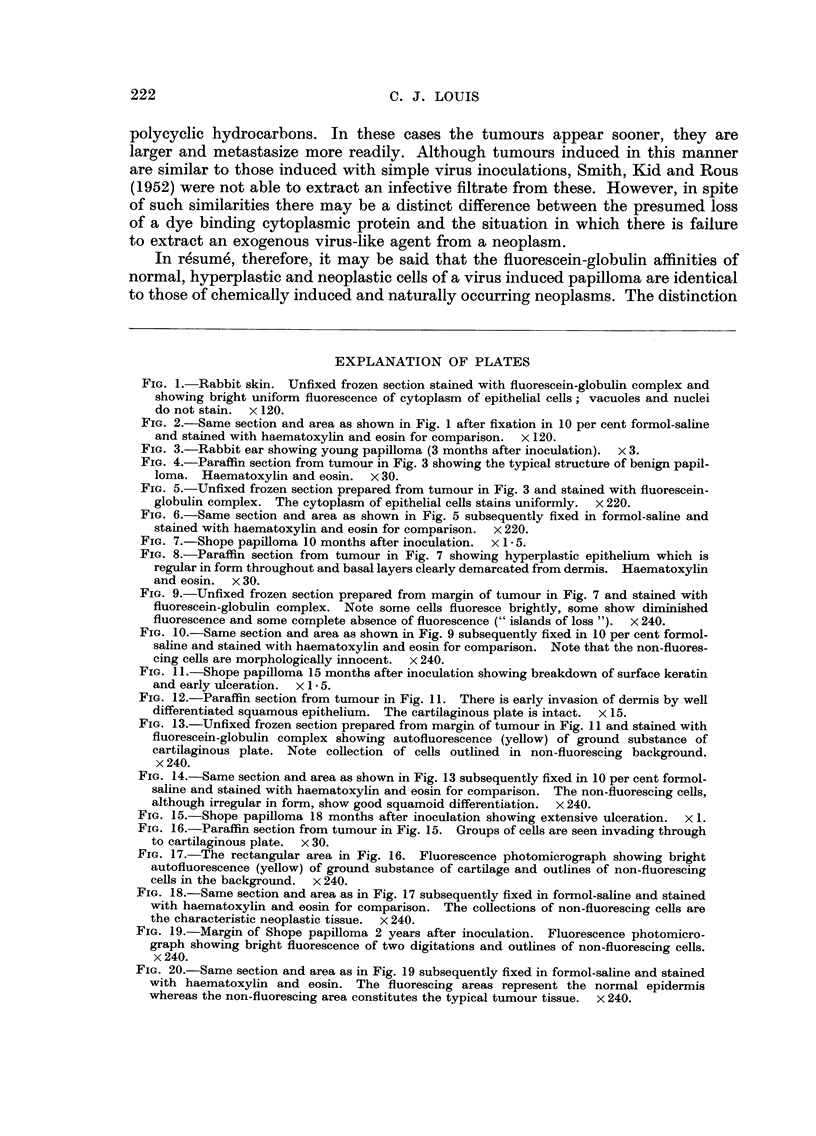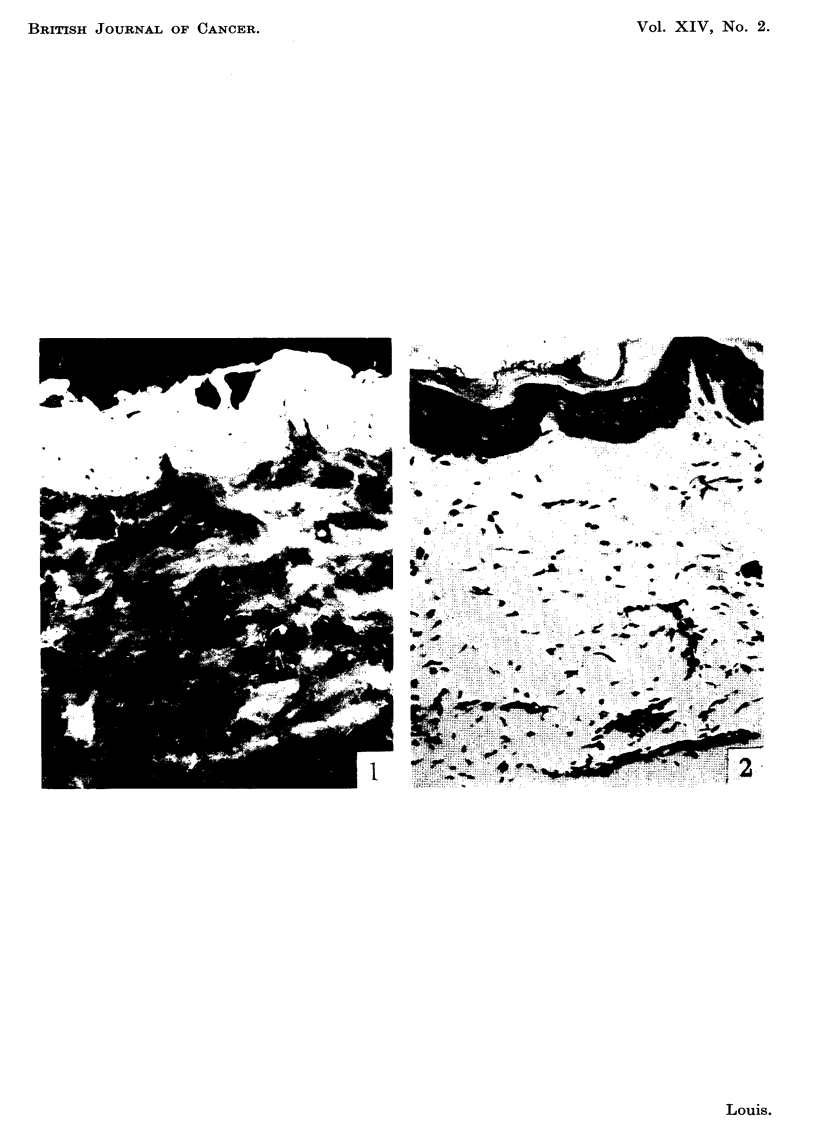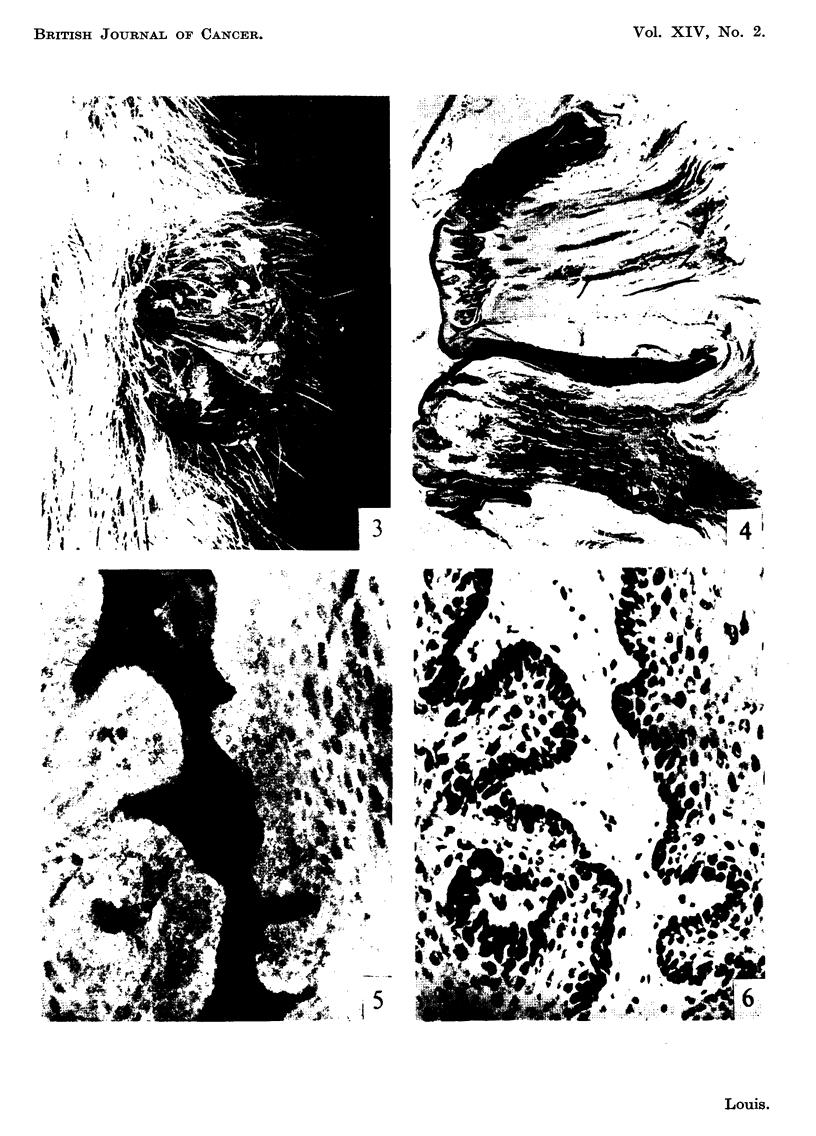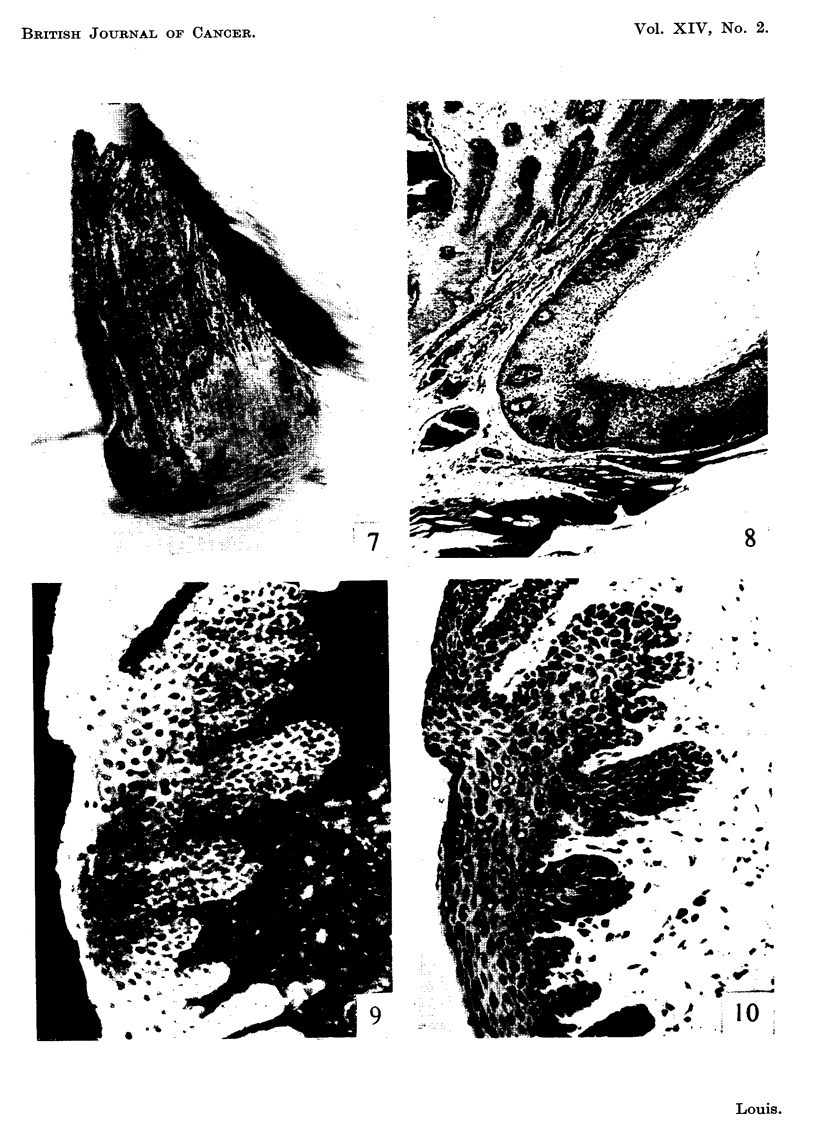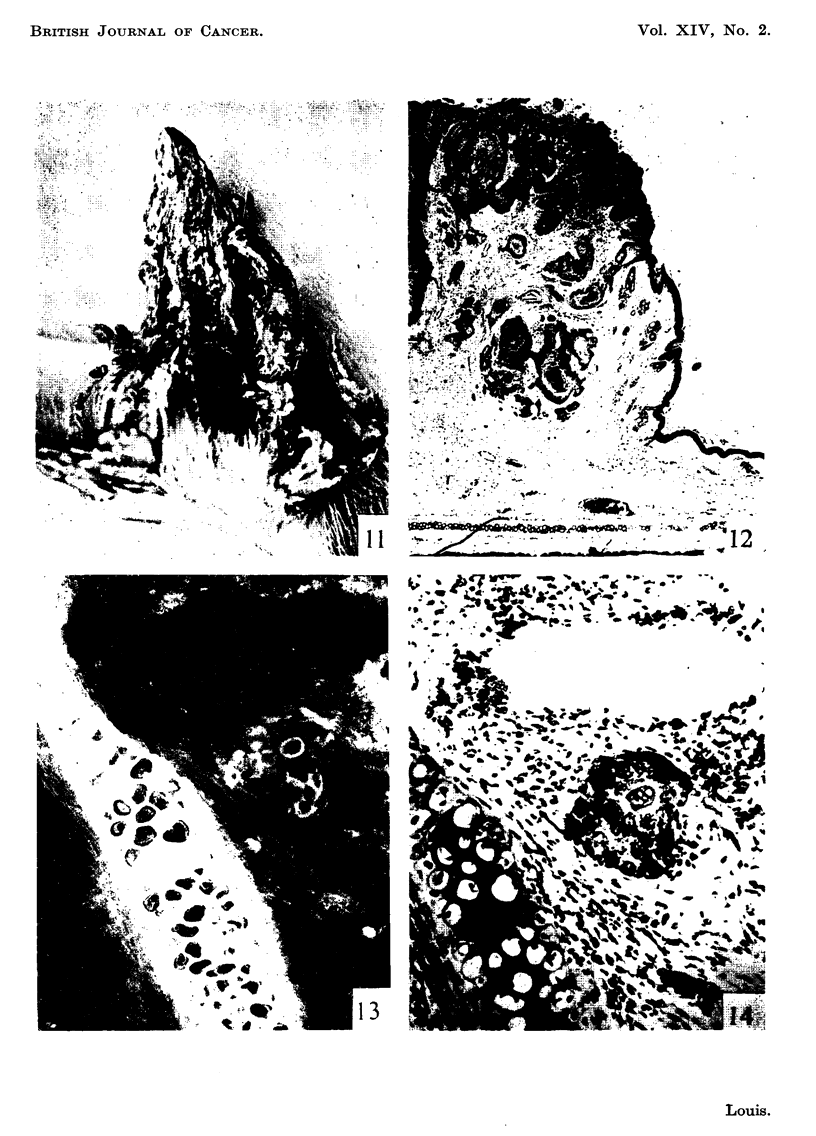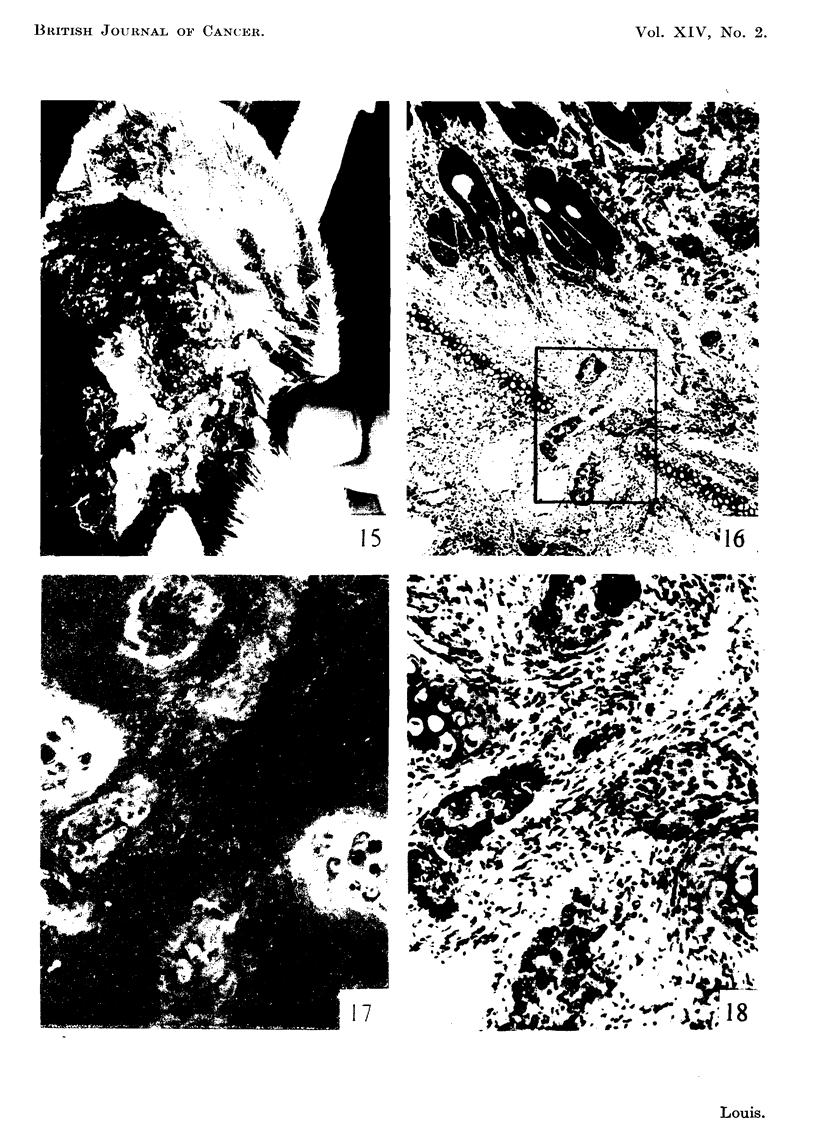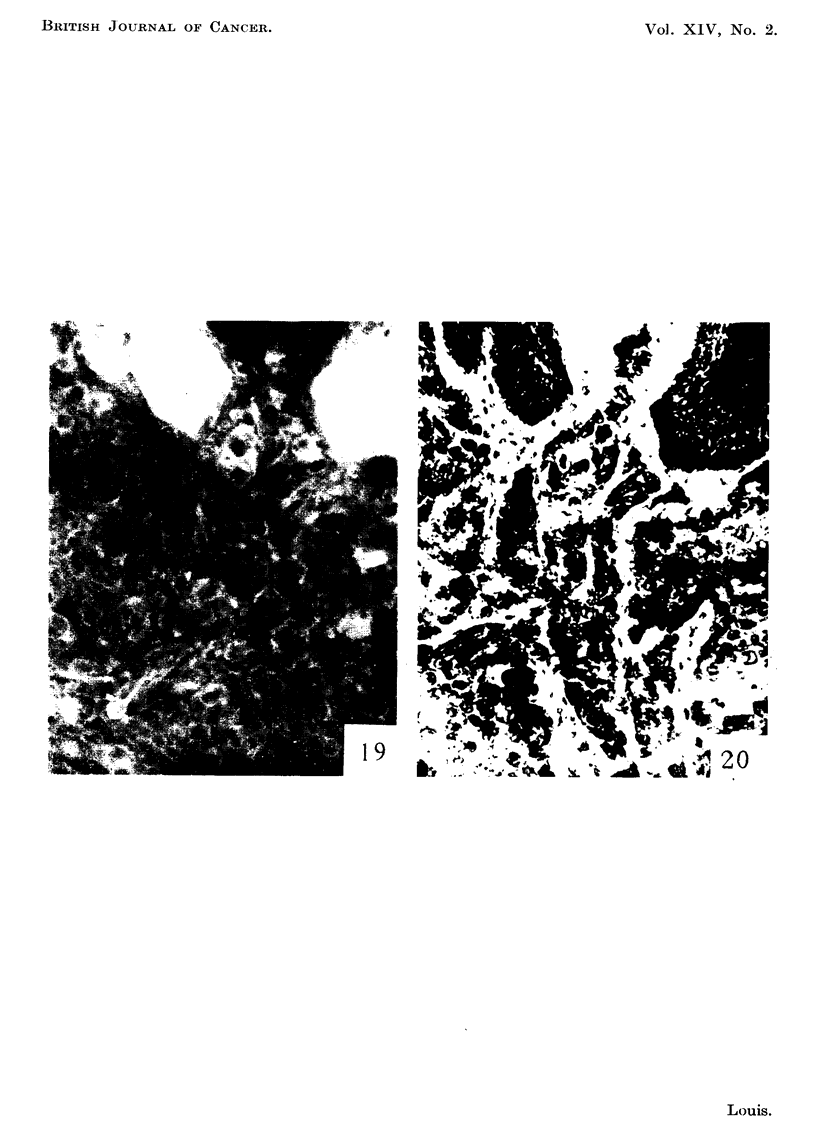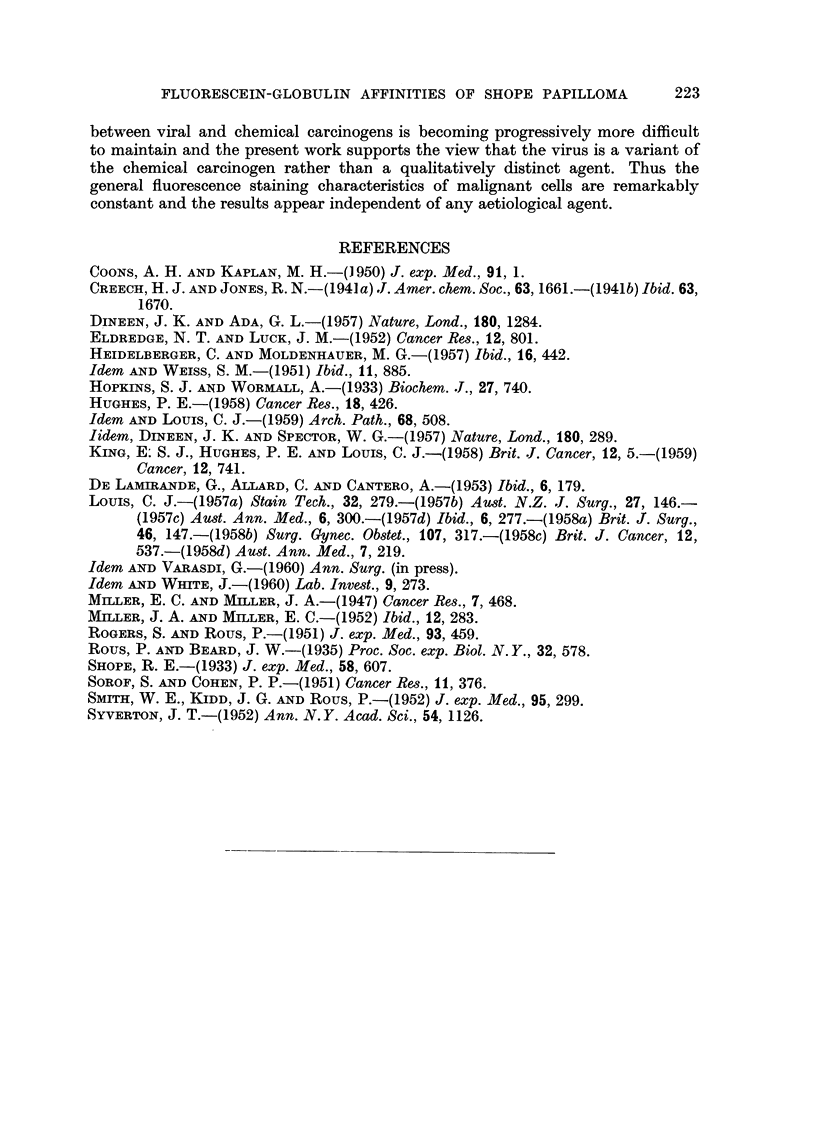# Fluorescein-globulin Affinities of the Shope Papilloma

**DOI:** 10.1038/bjc.1960.27

**Published:** 1960-06

**Authors:** C. J. Louis

## Abstract

**Images:**


### FLUORESCEIN-GLOBULIN AFFINITIES OF THE SHOPE PAPILLOMA

C. J. LOUIS

*From the Department of Pathology, University of Melbourne, Melbourne, Australia*

Received for publication April 30, 1960

THE fluorescein-globulin method of staining which distinguishes neoplastic from non-neoplastic cells has now been employed in a sufficiently large number of cases to demonstrate that it applies consistently both to chemically induced tumours in animals and to naturally occurring tumours in both animals and man (Louis, 1957*b*, 1958*a*, 1958*b* ; Louis and Varasdi, 1960). The test also affords a means of differentiating acute leukaemia from the chronic form (Louis, 1957*c*, 1958*d*).

It has been shown that all normal tissue cells of vertebrates and some invertebrates stain well and fluoresce brightly provided they contain an adequate amount of cytoplasm. The few exceptions that do not stain, namely : resting connective tissue cells, red blood cells, cells of the central nervous system and cells in tissue culture, have been discussed previously (Louis, 1958*c* ; Louis and White, 1960). The significance of the staining reaction although not yet completely understood has been attributed to protein-protein interactions between the basic cytoplasmic protein(s) of the normal tissue cells and the labelled serum proteins which have been rendered less basic by the conjugation process (Creech and Jones, 1941*a*, 1941*b* ; Hopkins and Wormall, 1933). That no serological implication is concerned in this reaction has been demonstrated by the observation that identical results could be obtained by using normal rabbit globulin (Hughes, Louis, Dineen and Spector, 1957), albumin and globulin fractions of many different species and even of the same animal (King, Hughes and Louis, 1958, 1959) and finally by egg albumen (Hughes and Louis, 1959). All malignant cells, on the other hand, even though containing large volumes of cytoplasm, failed to stain. Concomitant with the onset of the malignant change tumour cytoplasmic proteins become less basic (Eldredge and Luck, 1952 ; de Lamirande, Allard and Cantero, 1953 ; Sorof and Cohen, 1951). Hence it is to be expected that these proteins would have a decreased capacity to form complexes with the fluorescein-globulin conjugates. This has been found to be the case in a large number of tumours examined. In addition, certain presumably " premalignant " but morphologically normal cells were found not to stain.

Because these observations relate only to chemically induced and naturally occurring tumours it was decided to investigate a virus induced tumour, partly to study virus infected cells and partly to see if this type of neoplastic change differs from the chemically induced variety. Thus the results of the staining affinities of the Shope papilloma during its course of development are presented and discussed.

### METHODS AND MATERIALS

### *Papilloma virus*

The virus for inoculation was supplied by Dr. Richard E. Shope of the Rockefeller Institute in tissue slices of freshly removed warts stored in 50 per cent. glycerol. The tumour slices were ground with washed sand and normal saline to yield a 10 per cent suspension which was subsequently filtered through filter paper (Whatman No. 1). This filtrate was used for inoculation.

### *Inoculation of rabbits*

Twenty-four young white rabbits of mixed breed had small areas shaved on the dorsum of the distal halves of both ears followed by light scarification with sand paper. Inoculation was effected in some by rubbing the virus suspension into these areas, whilst in others by intradermal injection of 0·1 ml.

A second group of 12 rabbits had similar areas of skin prepared which were painted daily 5 days per week for approximately 12–14 weeks with the following carcinogens: 3 : 4-benzpyrene, 1 : 2 : 5 : 6-dibenzanthracene and 20-methylcholanthrene. These carcinogens were made up in 0·2 per cent solution with benzene. When small wart-like projections appeared the carcinogen application was stopped and each rabbit was given 2·0 ml. of the infected filtrate intravenously.

### *Production of hepatomata*

Adult Sprague Dawley rats weighing 150–200 g. were used. Hepatomata were induced by feeding 0·06 per cent 4-dimethylaminoazobenzene in a 20 per cent casein diet containing 2 mg. of riboflavine per kg. Rats were killed under ether anaesthesia at 2 weeks and then at weekly intervals until 12-15 weeks when liver tumours appeared. These have been described in detail elsewhere (Hughes, 1958 ; Hughes, Louis, Dineen and Spector, 1957) and are used here for comparison with the rabbit tumours (Table I).

### *Preparation of sera*

The globulin fraction was extracted from the serum of both inoculated and non-inoculated rabbits by precipitation with half saturated ammonium sulphate and after dialysis was conjugated with fluorescein isocyanate (Isomer I) by the method of Coons and Kaplan (1950).

### *Preparation of tissue sections*

In most cases small pieces of biopsy material were taken from the margins of the tumours and immediately snap frozen by dropping in isopentane precooled to $-75°$ C. in an ethanol-dry ice mixture. From these unfixed frozen sections were cut at 5–7 $\mu$ by a method previously described (Louis, 1957*a*).

### *Fluorescence staining, microscopy and photography*

Before staining, the free fluorescein derivatives were removed from the conjugated sera with ethyl acetate (Dineen and Ada, 1957). The dried tissue sections were treated with the labelled globulins for 10 minutes at room temperature, washed gently in three changes of buffered saline, pH 7·3, and examined in ultraviolet and blue light with a Leitz fluorescence microscope. Appropriate areas were selected and photographed using ultra-violet light, then the same sections were fixed in 10 per cent formol-saline, stained with haematoxylin and eosin and, for comparison, the same areas were rephotographed using visible light.

### RESULTS

The macroscopic and microscopic observations on papillomatosis experimentally induced in this series of animals were similar in all cases and good accounts have been given by Shope (1933), Rous and Beard (1935) and Syverton (1952). For the purpose of this study the development of these cutaneous tumours is treated in four stages :

(*a*) The phase of active epidermal proliferation ;
(*b*) the quiescent phase ;
(*c*) a premalignant phase ; and
(*d*) the malignant phase.

Unfixed frozen sections prepared from all tissues investigated were examined first in the unstained state to exclude any intrinsic fluorescence present independently of the use of the stain. As with previous experiments it was observed that elastic tissue and the ground substance of cartilage emit a bright yellow autofluorescence and keratin pale blue. A particular effort was made to avoid confusing these with the characteristic green fluorescence which results after staining with the fluorescein-labelled sera.

### *Normal rabbit epidermis*

All the cellular layers of the stratified squamous epithelium in rabbit skin showed a strong affinity for fluorescein-globulin complexes and emitted a bright green fluorescence in ultra-violet light (Fig. 1 and 2). There was a uniform distribution of the dye within the cells in which the cytoplasm, but not the nucleus, fluoresced. The cells lining the hair follicles and sudoriferous glands also stained uniformly.

### *Proliferating epidermis*

This stage of development became apparent macroscopically between the 10th and 14th day after inoculation as small papular vesicles which steadily progressed and developed into large verrucous masses (Fig. 3). This phase lasted approximately 5 months. Microscopically, in their well developed stage, the warts showed considerable thickening of the epidermis with papillary outgrowths from the surface and gross keratin formation (Fig. 4). There was active proliferation of the prickle cell layer with increase in the number of cells and numerous mitotic figures. Many of these cells contained pigment. These cells, however, were regular in form and the deeper layers clearly demarcated from the underlying connective tissue stroma. Small collections of wandering cells, principally of the small round cell type, were present in the superficial dermis.

Histochemically, after staining with the conjugated dye, the hyperplastic cells and those which were undergoing mitosis fluoresced brightly and uniformly as normal epithelium (Fig. 5 and 6).

*Premalignant phase*

About 6 months after inoculation the growths stopped growing macroscopically which was evidenced microscopically by the gradual decrease in the number of cells, absence of mitotic figures and desquamation of the superficial epidermal layers. This persisted for a further 6 months, during which period some involution took place (Fig. 7 and 8).

The fluorescence staining characteristics in the beginning of this phase were similar to those observed with normal and hyperplastic epithelium—namely, a uniform staining of the epithelial cells. At the 10th month there were observed groups of epithelial cells which showed a diminished affinity for the stain and failed to fluoresce in ultra-violet light (Fig. 9 and 10). These islands varied in size and contained anything from 6 up to 40 cells. No morphological evidence of malignancy was detected when these sections were stained with a routine stain such as haematoxylin and eosin. Again the cells were regular in form throughout and the basal layers sharply demarcated from the underlying stroma.

*Malignant phase*

Irregular " islands of loss " of staining were observed until 15 months without histological evidence of any malignant development. At about this time evidence of invasion became apparent. Masses of cells, arranged in small groups and strands and showing epidermoid differentiation, extended into the underlying stroma. These were irregular in shape, size and nuclear densities and showed many mitotic figures. Initially these were confined to the superficial dermis (Fig. 11–14). Later collections of cells were seen invading the cartilaginous plate (Fig. 15–18) and finally penetrated through the ventral aspect of the rabbits' ears. Here a clear-cut difference was observed between the non-fluorescing invading tumour tissue and the brightly fluorescing invaded ventral epidermis (Fig. 19 and 20).

*Exposure to chemical carcinogens*

In rabbit ears pretreated with carcinogens similar microscopic and macroscopic changes were observed but the malignant transformation after intravenous injection of the virus occurred much earlier than in the first group. The epidermal cells showed strong affinities for both conjugated dyes up to the 6th month stage of development when islands of loss of staining could be demonstrated. These islands persisted only for 3 months before obvious malignant changes became apparent.

The resultant tumours appeared more active than the previous ones in that invasion was much more rapid and metastases more widespread.

In sections taken from both primary and secondary nodules no fluorescence was emitted by the tumour cell cytoplasm.

*Rat liver after 4-dimethylaminoazobenzene administration*

Examination of sections from the livers of rats fed 4-dimethylaminoazobenzene showed that all the parenchymal cells stained uniformly up to the 3rd week of carcinogen administration. After this irregular islands of morphologically normal parenchymal liver cells were observed not to stain. These islands persisted for 12–15 weeks when hepatomata developed. All the tumour cells in these growths failed to stain with the labelled sera in contrast to the brightly fluorescing adjacent normal liver cells.

### DISCUSSION

Although numerous studies on the nature of neoplasia have been made and much information collected, an understanding of the fundamental processes involved is still distant. During the last century numerous vague aetiological factors were held responsible for the neoplastic change but these have recently become replaced by a number of distinct agents, which have been subdivided into chemical, physical ($\beta$-, $\gamma$-, and related rays) and organismal (particularly the viruses).

Much of our present knowledge of carcinogenesis has stemmed from the discovery and study of the chemical carcinogens. To date many such substances have been discovered but it has been shown that often a carcinogen will affect only a particular organ of a particular species. This effect may be produced by local application, oral administration or by injection parentally. Repeated observations with three groups of substances, namely: aminoazo dyes (Miller and Miller, 1947), aromatic polycyclic hydrocarbons (Heidelberger and Moldenhauer, 1957; Heidelberger and Weiss, 1951) and aminofluorenes (Miller and Miller, 1952), have shown that the carcinogen is taken up by the cells of the susceptible tissue. Differential high speed centrifugation of the tissue homogenates and subsequent electrophoresis (Eldredge and Luck, 1952; de Lamirande, Allard and Cantero, 1953; Sorof and Cohen, 1951) indicated that the carcinogen became firmly bound to a protein or protein complex present in the soluble fraction of the cell cytoplasm. Investigation of the tumours which developed subsequently showed that the tumour cells not only lacked this dye but also the protein(s) which was present in the parent cell and to which the carcinogen presumably became attached (Miller and Miller, 1947). Thus there was a clearly defined and easily reproducible difference between a normal and a malignant cell. This difference has been demonstrated histochemically in a variety of naturally occurring and chemically induced tumours using fluorescein-globulin conjugates (Louis, 1957*b*; Louis, 1958*a*; Louis, 1958*b*; Louis and Varasdi, 1960). The normal cells, which contained the basic protein(s), reacted with the labelled dye and emitted a bright green fluorescence in ultra-violet light whereas the malignant counterparts did not. In most sections prepared from the livers of rats fed 4-dimethylaminoazobenzene non-staining islands of cells were seen. The failure of these islands of morphologically normal but perhaps premalignant parenchymal cells to fluoresce appeared to be of great aetiological significance.

Our knowledge of virus carcinogenesis, on the other hand, is relatively meagre. Difficulties encountered in extraction and purification of these viruses are necessarily responsible for the poor understanding of this group of neoplasms. Since the Shope papilloma virus was readily available and provided a good example of the relationship of viral agents to benign and malignant growths it has been studied in detail in the present paper. This virus, like the chemical carcinogens, has been shown to possess strong cytotropism causing only epidermal cells of rabbits to proliferate and, after a latent period, to become malignant (Shope, 1933). In the present investigation examination of many examples of epidermal hyperplasia which had progressed to the twelve month stage of development showed strong affinities for the conjugated dyes and fluoresced as brightly as the adjacent normal epidermis. This was a normal reaction and conformed with those made on naturally occurring hyperplastic conditions in man, on the early stages of chemically induced skin tumours in mice (Louis, 1958*b*) and on the hyperplastic tissue of the regenerating rat liver (Louis, 1957*d*). All forms of obviously malignant tissue in this series failed to fluoresce. This was particularly striking in the early stages where invasion was confined to the superficial dermis. Here groups of cells showing good squamoid differentiation with well-developed keratin nests and intercellular prickles lacked all affinities for the fluorescent stain and failed to show a positive staining reaction (Fig. 11–18). Here also islands of morphologically normal epidermal cells have been observed which have shown the characteristic loss of staining (Fig. 7–10). The persistence of these islands, their possession of fluorescence staining characteristics similar to those of frank epidermoid carcinoma cells and the subsequent development of carcinoma at these sites suggests strongly that they are premalignant foci.

In all tumours, both experimentally induced and naturally occurring, examined by this method of study, the difference from normal tissue has been clear-cut and well defined. Since this difference appears to be due to the absence from the malignant cell of a protein complex, it seems probable that such a change would not necessarily be an abrupt one. Indeed, the present and previous investigations indicate that the final form of tumours is due to a series of changes. The nature of such changes occurring in the cells during the preneoplastic phase and concomitant with the development of malignancy is still unknown. The first histochemical evidence that a change has occurred is indicated by the appearance of persistent islands of morphologically normal cells with a diminished affinity for fluorescein-globulin complexes, a feature common to both chemically and viral induced tumours. Thus if the changes occurring during viral carcinogenesis are compared with those induced with a chemical carcinogen such as the aminoazo dyes (Table I) the changes leading to malignancy are found to be similar. Furthermore, the Shope virus can act synergistically with certain carcinogenic hydrocarbons (Rogers and Rous, 1951). In the present investigation, this phenomenon has been demonstrated with 20-methylcholanthrene, 3 : 4-benzpyrene and 1 : 2 : 5 : 6-dibenzanthracene. Intravenous injection of papilloma virus induces malignant tumours in areas of skin which have previously been treated with

TABLE I.—*Comparison of Fluorescence Staining of Shope Rabbit Tumours and Hepatomata Induced in Rats by 4-Dimethylaminoazobenzene*

| | Shope virus | 4-DAB |
|---|---|---|
| Species specificity | Rabbit | Rat. |
| Organ specificity | Epidermis | Liver. |
| Presence of carcinogen in normal cells | Can be extracted from tumours up to 9 months after inoculation | Firmly bound to soluble cytoplasmic proteins for 9–12 weeks. Tissue turns pink on acidification. |
| " Islands of loss of fluorescence staining " of morphologically innocent cells | Detected between 12th–15th month after inoculation and before development of frank malignancy | Detected 4–12 weeks after feeding 4-DAB. |
| Presence of carcinogen in tumour cells | Malignant change occurs at approximately 18 months. No virus extracted from tumour tissue. Tumour cells fail to fluoresce | Hepatomas appear at approximately 12 weeks. Tumour tissue does not turn pink on acidification. Tumour cells do not fluoresce. |

polycyclic hydrocarbons. In these cases the tumours appear sooner, they are larger and metastasize more readily. Although tumours induced in this manner are similar to those induced with simple virus inoculations, Smith, Kid and Rous (1952) were not able to extract an infective filtrate from these. However, in spite of such similarities there may be a distinct difference between the presumed loss of a dye binding cytoplasmic protein and the situation in which there is failure to extract an exogenous virus-like agent from a neoplasm.

In résumé, therefore, it may be said that the fluorescein-globulin affinities of normal, hyperplastic and neoplastic cells of a virus induced papilloma are identical to those of chemically induced and naturally occurring neoplasms. The distinction

---

EXPLANATION OF PLATES

FIG. 1.—Rabbit skin. Unfixed frozen section stained with fluorescein-globulin complex and showing bright uniform fluorescence of cytoplasm of epithelial cells ; vacuoles and nuclei do not stain. ×120.

FIG. 2.—Same section and area as shown in Fig. 1 after fixation in 10 per cent formol-saline and stained with haematoxylin and eosin for comparison. ×120.

FIG. 3.—Rabbit ear showing young papilloma (3 months after inoculation). ×3.

FIG. 4.—Paraffin section from tumour in Fig. 3 showing the typical structure of benign papilloma. Haematoxylin and eosin. ×30.

FIG. 5.—Unfixed frozen section prepared from tumour in Fig. 3 and stained with fluorescein-globulin complex. The cytoplasm of epithelial cells stains uniformly. ×220.

FIG. 6.—Same section and area as shown in Fig. 5 subsequently fixed in formol-saline and stained with haematoxylin and eosin for comparison. ×220.

FIG. 7.—Shope papilloma 10 months after inoculation. ×1·5.

FIG. 8.—Paraffin section from tumour in Fig. 7 showing hyperplastic epithelium which is regular in form throughout and basal layers clearly demarcated from dermis. Haematoxylin and eosin. ×30.

FIG. 9.—Unfixed frozen section prepared from margin of tumour in Fig. 7 and stained with fluorescein-globulin complex. Note some cells fluoresce brightly, some show diminished fluorescence and some complete absence of fluorescence (" islands of loss "). ×240.

FIG. 10.—Same section and area as shown in Fig. 9 subsequently fixed in 10 per cent formol-saline and stained with haematoxylin and eosin for comparison. Note that the non-fluorescing cells are morphologically innocent. ×240.

FIG. 11.—Shope papilloma 15 months after inoculation showing breakdown of surface keratin and early ulceration. ×1·5.

FIG. 12.—Paraffin section from tumour in Fig. 11. There is early invasion of dermis by well differentiated squamous epithelium. The cartilaginous plate is intact. ×15.

FIG. 13.—Unfixed frozen section prepared from margin of tumour in Fig. 11 and stained with fluorescein-globulin complex showing autofluorescence (yellow) of ground substance of cartilaginous plate. Note collection of cells outlined in non-fluorescing background. ×240.

FIG. 14.—Same section and area as shown in Fig. 13 subsequently fixed in 10 per cent formol-saline and stained with haematoxylin and eosin for comparison. The non-fluorescing cells, although irregular in form, show good squamoid differentiation. ×240.

FIG. 15.—Shope papilloma 18 months after inoculation showing extensive ulceration. ×1.

FIG. 16.—Paraffin section from tumour in Fig. 15. Groups of cells are seen invading through to cartilaginous plate. ×30.

FIG. 17.—The rectangular area in Fig. 16. Fluorescence photomicrograph showing bright autofluorescence (yellow) of ground substance of cartilage and outlines of non-fluorescing cells in the background. ×240.

FIG. 18.—Same section and area as in Fig. 17 subsequently fixed in formol-saline and stained with haematoxylin and eosin for comparison. The collections of non-fluorescing cells are the characteristic neoplastic tissue. ×240.

FIG. 19.—Margin of Shope papilloma 2 years after inoculation. Fluorescence photomicrograph showing bright fluorescence of two digitations and outlines of non-fluorescing cells. ×240.

FIG. 20.—Same section and area as in Fig. 19 subsequently fixed in formol-saline and stained with haematoxylin and eosin. The fluorescing areas represent the normal epidermis whereas the non-fluorescing area constitutes the typical tumour tissue. ×240.

Louis.

3

4

5

6

7

8

9

10

11

12

13

14

Louis.

15

16

17

18

Louis.

19

20

between viral and chemical carcinogens is becoming progressively more difficult to maintain and the present work supports the view that the virus is a variant of the chemical carcinogen rather than a qualitatively distinct agent. Thus the general fluorescence staining characteristics of malignant cells are remarkably constant and the results appear independent of any aetiological agent.

### REFERENCES

COONS, A. H. AND KAPLAN, M. H.—(1950) *J. exp. Med.*, **91**, 1.
CREECH, H. J. AND JONES, R. N.—(1941*a*) *J. Amer. chem. Soc.*, **63**, 1661.—(1941*b*) *Ibid.* **63**, 1670.
DINEEN, J. K. AND ADA, G. L.—(1957) *Nature, Lond.*, **180**, 1284.
ELDREDGE, N. T. AND LUCK, J. M.—(1952) *Cancer Res.*, **12**, 801.
HEIDELBERGER, C. AND MOLDENHAUER, M. G.—(1957) *Ibid.*, **16**, 442.
*Idem* AND WEISS, S. M.—(1951) *Ibid.*, **11**, 885.
HOPKINS, S. J. AND WORMALL, A.—(1933) *Biochem. J.*, **27**, 740.
HUGHES, P. E.—(1958) *Cancer Res.*, **18**, 426.
*Idem* AND LOUIS, C. J.—(1959) *Arch. Path.*, **68**, 508.
*Iidem*, DINEEN, J. K. AND SPECTOR, W. G.—(1957) *Nature, Lond.*, **180**, 289.
KING, E. S. J., HUGHES, P. E. AND LOUIS, C. J.—(1958) *Brit. J. Cancer*, **12**, 5.—(1959) *Cancer*, **12**, 741.
DE LAMIRANDE, G., ALLARD, C. AND CANTERO, A.—(1953) *Ibid.*, **6**, 179.
LOUIS, C. J.—(1957*a*) *Stain Tech.*, **32**, 279.—(1957*b*) *Aust. N.Z. J. Surg.*, **27**, 146.—(1957*c*) *Aust. Ann. Med.*, **6**, 300.—(1957*d*) *Ibid.*, **6**, 277.—(1958*a*) *Brit. J. Surg.*, **46**, 147.—(1958*b*) *Surg. Gynec. Obstet.*, **107**, 317.—(1958*c*) *Brit. J. Cancer*, **12**, 537.—(1958*d*) *Aust. Ann. Med.*, **7**, 219.
*Idem* AND VARASDI, G.—(1960) *Ann. Surg.* (in press).
*Idem* AND WHITE, J.—(1960) *Lab. Invest.*, **9**, 273.
MILLER, E. C. AND MILLER, J. A.—(1947) *Cancer Res.*, **7**, 468.
MILLER, J. A. AND MILLER, E. C.—(1952) *Ibid.*, **12**, 283.
ROGERS, S. AND ROUS, P.—(1951) *J. exp. Med.*, **93**, 459.
ROUS, P. AND BEARD, J. W.—(1935) *Proc. Soc. exp. Biol. N.Y.*, **32**, 578.
SHOPE, R. E.—(1933) *J. exp. Med.*, **58**, 607.
SOROF, S. AND COHEN, P. P.—(1951) *Cancer Res.*, **11**, 376.
SMITH, W. E., KIDD, J. G. AND ROUS, P.—(1952) *J. exp. Med.*, **95**, 299.
SYVERTON, J. T.—(1952) *Ann. N.Y. Acad. Sci.*, **54**, 1126.